# Prevalence and risk factors for non-collision injuries among bus commuters in Dar es Salaam, Tanzania

**DOI:** 10.1186/s12889-022-13284-9

**Published:** 2022-05-13

**Authors:** Alan Lwanga, Hussein H. Mwanga, Ezra J. Mrema

**Affiliations:** 1 Digital Adherence Research Department, KNCV Tuberculosis Foundation, Dar es Salaam, Tanzania; 2 Department of Environmental and Occupational Health, School of Public Health and Social Sciences, Muhimbili University of Health and Allied Sciences, Dar es Salaam, Tanzania

**Keywords:** Non-collision injuries, Prevalence, Commuters, Dar es Salaam, Tanzania

## Abstract

**Background:**

Migration of people from rural to urban areas has led to the increase in demand for transportation services in the cities. However, authorities have failed to cope with this problem in a consistently manner. This has led to the increase in non-collision injuries among commuters. This study aimed at investigating the prevalence and risk factors for non-collision injuries among commuters using public transport in Dar es Salaam, Tanzania.

**Methods:**

A cross sectional study was conducted in Dar es Salaam involving 290 commuters from 7 bus routes travelling to and from the city centre using public transport which are privately owned and commonly known as “*daladala*”. Stratified random sampling was used to sample buses based on the passenger carrying capacity (i.e. 15-24, 25-34 and 35-45 passengers). Systematic random sampling was used to get a total of three commuters from each bus for the interview.

**Results:**

Lifetime prevalence of non-collision injuries was 71%, while these rates were 70 and 39% in the last 12 and 6 months, respectively. Commuters aged between 18 and 28 years experienced non-collision injuries the most (56%) in the last 12 months. Most non-collision injuries occurred on weekdays in the evening from 5:00 pm to 10:00 pm. Strong association was observed between the occurrence of non-collision injuries and commuting time between 5:00 pm to 10:00 pm (adjusted OR = 9.24; 95% CI: 2.68-19.54); boarding and disembarking (adjusted OR = 9.21; 95% CI: 3.77-25.11) and scrambling during boarding (adjusted OR = 5.03; 95% CI: 2.51-21.32). The lower limbs (adjusted OR = 8.64; 95% CI: 2.72-21.76) and the upper limbs (adjusted OR = 13.55; 95% CI: 5.32-33.21) were the most affected body parts.

**Conclusions:**

This study has demonstrated high prevalence of non-collision injuries among commuters using public transport in Dar es Salaam. Travelling in the evening between 5:00 pm to 10:00 pm on the weekdays, boarding and disembarking especially when scrambling for the bus during boarding, overcrowding in the bus especially when the bus is already full with no seats available are the major risk factors for non-collision injuries.

## Background

Commuters using public transport in urban areas experience different challenges such as non-collision injuries, discomfort, overcrowding and stress while on transit. Non-collision injuries are sustained by commuters in incidents where there is no vehicle collision [1]. According to social ecological model for unintentional injuries, non-collision injuries occur as a result of an interaction between several factors [2]. These include individual factors (such as scrambling during boarding); situational factors (such as overcrowding or time of day/week) and institutional factors. Institutional factors relate to lack of enforcement for good driving standards, and bus interior settings such as higher kerb height, slippery floor and unsafe interior following modification for use as commercial vehicles with no standardized safety guidelines [3]. Institutional factors also include investment in public transport infrastructures, urban planning as well as policies related to urban planning to public transport, however, these are beyond scope of this study. In a nutshell, non-collision injuries experienced by commuters are in fact unintended consequences of using public transport.

Globally, several studies have been conducted on non-collision injuries among commuters using public transport. A prevalence of 58 and 64% for non-collision injuries was reported in different studies in Israel and United Kingdom, respectively [4–6]. Most body parts injured include head, chest, upper and lower limbs [7]. In Africa, studies indicate that non-collision injuries are common among commuters above the age of 60 years and mostly occur on weekdays [4]. Furthermore, in South Africa, commuters travelling using public buses experience mostly minor injuries, on average, in non-collision incidences compared to collision incidences where most injuries are serious. In Tanzania, studies on non-collision injuries with minor severity levels among women reported a prevalence of 46% [8]. In a nutshell, women had a higher risk of experiencing non-collision injuries when using public transport compared to men. Although the study reports the non-collision injuries prevalence for both males and females, it does not explicitly characterise the injuries by the body parts affected. An assessment of public transport in Dar es Salaam, Tanzania revealed failure of regulatory authorities to manage physical bus stand infrastructures and inability enforce regulations were a major pre-disposing factor for injuries among commuters [9].

In Tanzania, Land Transport Regulatory Authority (LATRA), formerly Surface and Marine Transport Regulatory Authority (SUMATRA) under the Ministry of Transport and Communication, is tasked to oversee, manage and regulate public transportation in the country [10]. Low capacity to enforce compliance and lack of coordinated efforts with other institutions such as Tanzania Road Agency (TANROADS) and Dar es Salaam city council have been identified as among the factors that exacerbate poor public transport in Dar es Salaam and consequently the increase in the incidence of non-collision injuries [10].

Given the fact that most non-collision injuries that occur in Dar es Salaam are underreported and generally considered individual matters, the study aimed at investigating prevalence of non-collision injuries among commuters, their characterization and associated factors such as age, gender, commuting time, overcrowding levels, injury mechanisms and commuters’ body position at the time of injury.

## Methods

This cross-sectional study was conducted in Dar es Salaam region in Tanzania. The region has five districts namely Kinondoni, Ilala, Temeke, Ubungo and Kigamboni. Commuters travelling in public buses from Kinondoni, Ilala, Temeke and Ubungo heading to and from the city centre were selected to participate in the study because of their high risk of sustaining non-collision injuries. Such high risk is mainly a result of the limited number of buses against the demand which makes commuters more likely to scramble during boarding the bus. The dependent variable of this study was whether a commuter had experienced a non-collision injury or not [11]. Independent variables included injury time period, bus carrying capacity, injury causation, type of body part injured and injury severity which were defined according to Kirk et al. [5]. This classification categorizes slight injuries as the ones that require no form of medical treatment or can be ignored, and serious injuries as the ones that involve obligatory medical treatment such as fractures, severe cuts as well as fatal injuries in which death might occur within 40 days of the injury. Age and gender were the confounding variables which were controlled for in multiple logistic regression models.

### Sampling

The study was conducted from June to September 2020. The study adopted a multistage sampling procedure to select bus routes, buses and commuters. The bus passenger carrying capacity according to SUMATRA (i.e. 15-24, 25-34 and 35-45 passengers) served as strata for each of the routes selected.

The study used probability sampling procedure for the proportion of non-collision injuries. The calculation of the sample size for a proportion (Z^2^p(1-p)/E^2^) of non-crash injuries among passengers (*p* = 64%) was adopted based on Kirk et al. [5]. This population category was selected by using a formula adopted from Israel [12], where 95% indicates a confidence level (Z) and 6% margin of error (E). The response rate of 85% indicated a sample size of 290 participants. The current study however is inclusive as it covers both men and women.

By using a systematic sampling approach, every third bus in a waiting queue for the given route in a major bus station was selected. Commuters’ participation was purely voluntary. They were selected by using systematic random sampling technique, where every fifth commuter to board the bus was requested to take part in the study. To avoid recruiting commuters with no experience with public transport, the study was restricted to commuters who are frequent users of public transport for at least a year. Both drivers and commuters were informed about the purpose of the study and their consent was requested. The selected major bus stations with buses operating to and from the city centre included Ubungo (i.e. Simu-2000), Kimara, Mbezi, Makumbusho, Mbagala, Tabata and Gongolamboto.

### Data collection

On board interviews with commuters were conducted by using a structured questionnaire that was pre-tested with commuters of Gongolamboto to Mbagala route. As such, this route was not part of the study. Pretesting of the tools was done to ensure the validity and reliability of the collected data. The questionnaire was used to gather information about socio-demographic characteristics, non-collision injuries experiences when using public transport, and other associated details about injury timing and mechanism of the injury. The questionnaire was divided into three parts. The first part addressed demographic information such as gender, age, place of origin and occupation. The second part addressed the non-collision injuries prevalence, characterization such as injury frequency and body part injured and other associated risk factors. The last part addressed the commuters perspective of measures for mitigating the problem. The questionnaire was prepared in English language, translated to Swahili and the response were translated to English. Prior to data collection, research assistants were trained and familiarized with the study objectives and the data collection tools. In each bus, three commuters were interviewed using a smart phone with android application (Kobo Collect) and hence allowing for some privacy and avoiding unnecessary distractions to other passengers. During consenting, participants were asked if they were comfortable responding to questions in a bus with other passengers and agreed to that process. The interviews were conducted in the morning and evening times of weekdays and weekends in order to maximize sample heterogeneity.

### Statistical analyses

The data was analysed by using R-studio software version 1.0.136 for both descriptive and inferential statistical approaches. The study used inferential statistical approaches namely chi-square test and logistic regression where *p*-values of less than 0.05 were considered statistically significant. The chi-square test was used to show the relationship between non-collision injury and independent categorical variables (i.e. injury time, bus carrying capacity, injury causation, type of body part injured and injury severity). Multiple logistic regression models were used to determine factors which were associated with non-collision injuries while correcting for confounders (i.e. age and gender).

## Results

### Demographics characteristics of respondents

A total of 296 commuters were contacted and 290 participated in the study. This gives a response rate of 98%. The median age of respondents was 27 years with IQR ranging between 23 and 33 years. Most commuters were males (64%). Majority of study participants had completed primary education (28%), secondary education (25%) and the university (25%) (Table 1).

**Table 1:** Demographic characteristics of commuters

Characteristic	Frequency (%)
**Age (in years)**	
Years [median (IQR)]	27 (23-33)
18-28	160 (55)
29-39	96 (33)
40-50	28 (10)
51-61	6 (2)
**Gender**	
Male	186 (64)
Female	104 (36)
**Level of education**	
Incomplete primary education	4 (1)
Completed primary education	78 (28)
Completed secondary education (form IV)	73 (25)
Completed secondary education (form VI)	12 (4)
Completed vocational training/Diploma	50 (17)
University graduate	73 (25)
**Route**^**a**^	
Gongolamboto	42 (15)
Kimara	41 (14)
Makumbusho	41 (14)
Mbagala	42 (14)
Mbezi	42 (15)
Tabata	41 (14)
Ubungo	41 (14)
**Bus passenger carrying capacity**	
25-34	96 (33)
35-45	95 (33)
46 and above	99 (34)
**District**	
Ilala	84 (29)
Temeke	40 (14)
Kinondoni	106 (37)
Ubungo	39 (13)
Kigamboni	10 (3)
Other^b^	12 (4)

^a^Include all buses routes heading to and from city center from the mentioned areas

^b^Other: Morogoro Manispaa, Chalinze, Kigoma Mjini, Karatu, Arumeru, Muheza, Simiyu and Bagamoyo

Non-collision injuries based on distribution of commuters from different bus routes in the study area are comparable (Table 1) similar to results on buses with different passengers capacity where each contributed a third of commuters in the study. Most commuters were from Kinondoni district (37%) followed by Ilala district (27%).

### Non-collision injury prevalence and characterization

Lifetime prevalence of non-collision injuries was 71%, while these rates were 70 and 39% in the last 12 and 6 months, respectively (Table 2). In addition, majority of commuters (97%) experienced between 1 to 4 non-collision injuries in the past 12 months.

**Table 2:** Prevalence and frequencies of non-collision injuries among commuters

**Prevalence Period**	**Prevalence (%)**
Lifetime	71
Within 12 months	70
Within 6 months	39
**Number of non-collision injuries**	**Frequency (%)**
1-2	163 (80)
3-4	35 (17)
5 and above	6 (3)
**Total**	**204 (100)**

### Non-collision injuries characterization

**Fig. 1:**
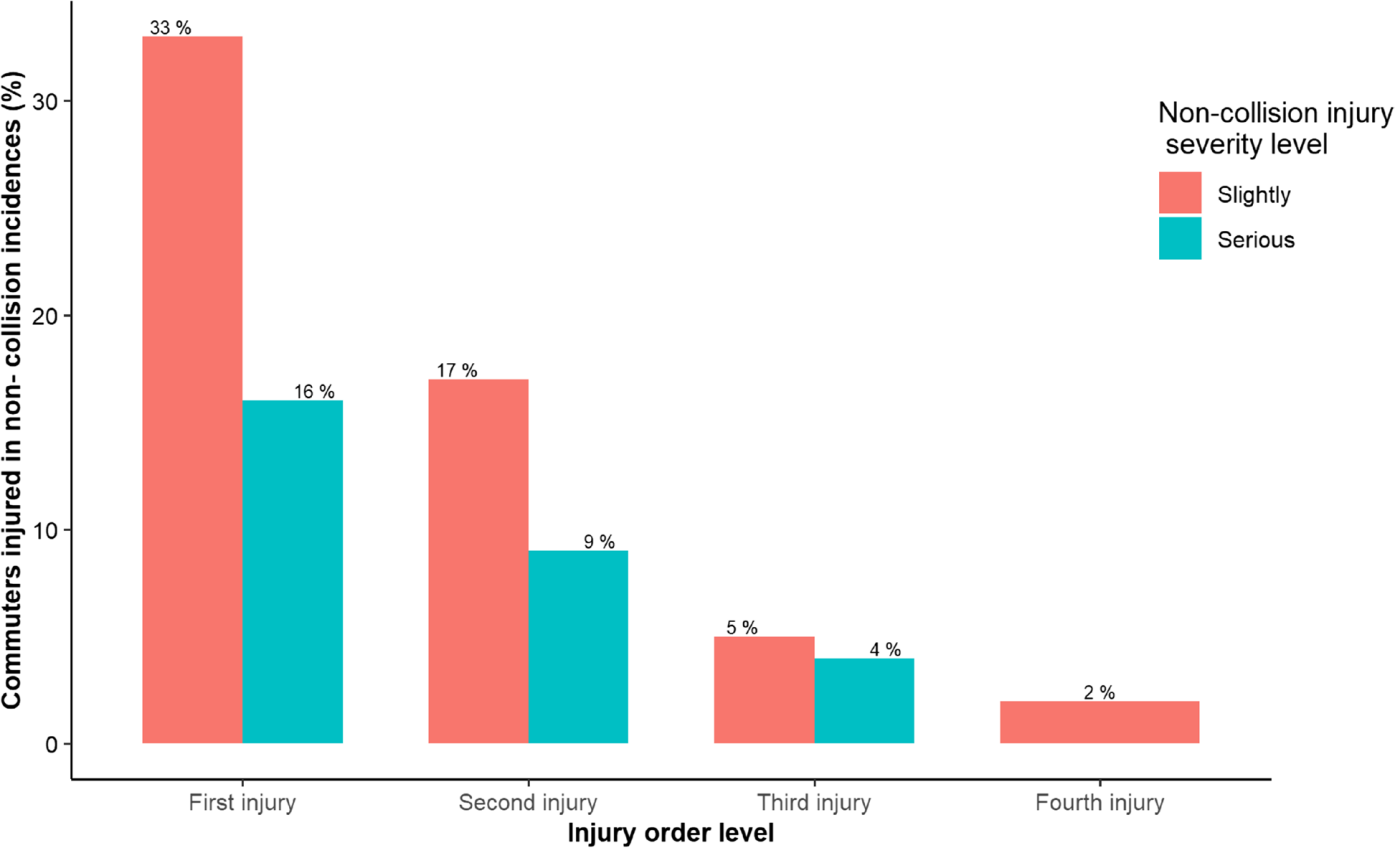
Non-collision injury severity on different injury frequencies

Results indicate that, males experience non-collision injuries the most (64%) compared to females (36%). Furthermore, commuters aged between 18 and 28 years experienced non-collision injuries the most (56%) followed by commuters aged between 29 and 39 years (34%) (Table 3). Most non-collision injuries were experienced on weekdays (82%), during the evening time between 5:00 pm-10:00 pm (36%) followed by the morning hours between 5:00 am-10:00 am (32%). Most non-collision injuries occurred while commuters were in the standing position (44%) and walking (37%). The most body parts injured were upper limbs (36%) and lower limbs (32%). On the other hand, those who boarded the bus while it is full experienced the most non-collision injuries (66%) with most occurred when scrambling (35%), boarding and disembarking (26%) (Table 3). The severity for majority of non-collision injuries was slightly serious with 33 and 17%, for the first and second injury, respectively (Fig. 1).

**Table 3:** Association between non-collision injuries occurrence and associated risk factors

Factor	Frequency (%)	COR (95% CI)	AOR (95% CI)
**Sex**			
Female	75 (36)		
Male	129 (64)	0.87 (0.51-1.48)	3.39 (0.76-6.18)
**Age (in years)**			
18-28	115 (56)	Reference	
29-39	69 (34)	0.87 (0.44-2.35)	3.55 (0.45-6.66)
40-50	15 (8)	0.45 (0.19-1.04)	0.41 (0.11-2.58)
51-61	5 (2)	1.76 (0.26- 4.75)	0.84 (0.15-4.5)
**Bus carrying capacity**			
25-34	96 (33)	Reference	
35-45	95 (33)	0.55 (0.28-1.05)	1.54 (0.37-3.19)
46 and above	99 (34)	2.87 (0.67-6.03)	3.65 (0.38-5.88)
**Injury time**			
05:00 am-10:00 am	106 (32)	Reference	
11:00 am-04:00 pm	100 (31)	0.88 (0.21-4.67)	0.59 (0.15-3.32)
05:00 pm-10:00 pm	119 (36)	***6.11 (2.27-18.61.)***	***9.24 (2.68-19.54)*** *
11:00 pm-04:00 am	2 (1)	0.82 (0.34-3.12)	1.47 (0.23-4.63)
**Injury mechanism(*n*** **= 426)**^**c**^			
Poor driving skills	57 (13)	Reference	
Boarding and disembarking	110 (26)	***6.36 (2.45-21.32)***	***9.21 (3.77-25.11)*** **
Scrambling^b^	148 (35)	***3.42 (1.92-14.19)***	***5.03 (2.51-21.32)*** **
Bus interior settings	111 (26)	7.43 (0.63-23.58)	4.07 (0.87-11.26)
**Weekly time(*n*** **= 390)**^**c**^			
Weekends	71 (18)	Reference	
Weekdays	319 (82)	***2.34(1.86-7.18)***	4.44 (2.43-9.63)***
**Body position(*n*** **= 244)**^**c**^			
Sitting	46 (19)	Reference	
Standing	108 (44)	***8.22 (3.01-22.92)***	4.84(1.28-12.11)***
Walking	90 (37)	***3.89 (1.32-7.55)***	4.78 (1.88-15.41)***
**Overcrowding status(*n*** **= 250)**^**c**^			
Seats available	29 (12)	Reference	
Standing room only	56 (22)	14.21 (0.32-22.76)	8.67 (0.46-15.31)
Already full when boarding	165 (66)	7.49 (2.22-18.32)	3.79 (1.55-9.72)
**Body part injured(*n*** **= 431)**^**c**^			
Stomach	16 (4)	Reference	
Face	25 (6)	1.19 (0.11-4.39)	0. 88 (0.25-4.61)
Lower limbs	138 (32)	***12.34 (3.03-27.11)***	8.64 (2.72-21.76)***
Head	60 (14)	2.44 (0.78-7.55)	1.15 (0.41-5.72)
Neck	27 (6)	0.34 (0.18-2.22)	0.86 (0.21-3.82)
Upper limbs	156 (36)	***9.87 (4.72-29.45)***	13.55 (5.32-33.21)***
Chest	9 (2)	0.33 (0.13-3.29)	1.01 (0.38-5.76)

Each OR represents a separate unadjusted logistic regression model

Bolded figures indicate statistical significant results

*COR* Crude Odds Ratio, *AOR* Adjusted Odds Ratio

^c^The results include the responses from all four injuries frequencies reported in which each factor is mutually inclusive which results to a different total n value for each factor

Adjusted for age and sex (confounders). *Scrambling during boarding means; non-collision injuries that occurred when a person was scrambling only

*Boarding and disembarking means; non-collision injuries that occurred when commuter is entering or stepping out of the bus without scrambling

*p*-value * < 0.05; ** < 0.01; *** < 0.001

### Relationship between non-collision injuries and risk factors

This study shows strong statistical evidence for an association between occurrence of non-collision injuries among commuters with commuting time between 5:00 pm and 10:00 pm (adjusted OR = 9.24; 95% CI: 2.68-19.54). Moreover, most non-collision injuries among commuters occurred during boarding and disembarking the bus (adjusted OR = 9.21; 95%CI: 3.77-25.11) especially when scrambling for the bus during boarding (adjusted OR = 5.03; 95% CI: 2.51-21.32). Lower limbs (adjusted OR = 8.64; 95%CI: 2.72 - 21.76) and upper limbs (adjusted OR = 13.55; 95%CI: 5.32-33.21) were the most affected body parts during non-collision injuries among commuters. Commuters were likely to experience non-collision injuries at a standing (adjusted OR = 4.84; 95% CI: 1.28-12.11) and walking (adjusted OR = 4.78; 95% CI: 1.88-15.41) body position. Furthermore, commuters travelling on weekdays (adjusted OR = 4.44; 95% CI: 2.43-9.63) were more likely to experience non-collision injuries compared to commuters travelling on weekends. Lastly, with regards to overcrowding, commuters boarding public buses when it is already full (adjusted OR = 3.79; 95% CI: 1.55-9.72) were more likely to experience non-collision injuries compared to other overcrowding status (Table 3).

## Discussion

The lifetime prevalence for non-collision injuries (71%) reported in this study was higher than what was reported by Bjornstig et al. [6], the only available previous study to have reported lifetime prevalence other than annual prevalence for these types of injuries among passengers in public transport buses. This is also a pushback to sustainable development goal 11 which emphasises on the need to make cities safe, resilient and sustainable by the year 2030 [13]. The annual prevalence for non-collision injury (70%) was also higher compared to those reported elsewhere [5–7]. This could be explained by the tendency of people to normalize scrambling for the public transport at boarding time [8]. Furthermore, the prevalence of non-collision injuries within the last six-month period (39%) is almost half the reported annual prevalence. The possible explanation for the reduction in non-collision injuries prevalence among commuters for 12 months and 6 months is thought to be associated with new restrictions imposed by the Tanzania government to combat spread of Covid-19 disease infections from March to June 2020. During this period, all public transport buses on transit were required to operate with seated passengers only [14].

### Social and economic factors with non-collision injuries

In the study, the annual prevalence of non-collision injuries was higher for males (64%) compared to females commuters (36%), contrary to the previous Tanzanian study [9]. In addition, studies suggest that males have a highest injury tolerance limit compared to females which suggest taking more risks like scrambling during boarding the bus could be the possible reason for the observed differences in the prevalence rates of non-collision injuries between them [5] Interestingly, this was different from studies reported in the UK and Sweden where 75 and 74% of all non-collision injuries were experienced by females, respectively [5, 6].

The commuters aged between 18 and 28 years experienced more non-collision injuries (56%) than commuters than any other age group. This was due to this age group association with scrambling during boarding the bus in rush hours compared to other age groups due to lack of experience and maturity [15]. This is different from experience in Sofia, Bulgaria by Zunjic et al. [7] and London in the United Kingdom [5] where majority of victims of non-collision were 60 years old and above [6]. This study revealed that, in all injury cases reported upper limbs were the most significantly injured part in non-collision incidences (36%) followed by the lower limbs (32%). This is also reported by Kanyama et al. [16] that, most bus conductors in Dar es Salaam tend to hurry commuters in stepping in and out of the bus, this made, most of the commuters to get injured at upper and lower limbs. The results are relatively similar to those reported in Israel where head (33%) and limbs (29%) were the most injured body parts of urban travellers during non-collision incidences [4, 6, 7].

### Non-collision injuries and its associated factors

Majority of non-collision injuries are caused by scramble when boarding the bus followed by injuries during boarding and disembarking. These are explained by a greater demand of transport services among commuters which does not match with the available services. This tend to force commuters to scramble for boarding and securing a seat or even a chance to travel within a pre-determined time instead of waiting for later buses. Conversely, the findings are different from the injury mechanisms reported in Israel where acceleration and deceleration by poor driving habits were the most causative source of non-collision injuries among commuters [4]. A similar study in Serbia reported that acceleration, boarding/disembarking was the major cause of non-collision injuries [7], while boarding and disembarking was the second most reason and it was comparable with other studies for the same reasons such as in United Kingdom [5], Sweden [6] and Serbia [7].

The current study found that majority of non-collision injuries occurred while commuters were in a standing position. This corroborates the findings of Silvano and Ohlin who suggested that passengers in a standing position during transit are more vulnerable to non-collision injuries due to their low ability to sustain balance when encountered by external force [16].

The findings indicated a statistical significant association between higher odds of occurrence of non-collision injuries and time between 05:00 pm to 10:00 pm compared to between 05:00 am and 10:00 am. This is explained by the lack of buses or delayed on arrivals of the public buses at the bus stations. Similar findings were also reported by Bjornstig et al. [6] who pointed out some statistical evidence on the relationship between the prevalence of non-collision injuries for travellers and rush hours especially in the morning and evening time.

Some statistical evidence suggests higher odds of occurrence of the non-collision injuries among commuters when boarding and disembarking compared to ones due to by bus driver’s poor driving skills. This is similar to findings reported by Odofuwa [17] who highlighted the difficulties in boarding vehicles contribution to non-collision injuries prevalence among elderly in Nigeria. Scrambling during boarding was another statistically significant non-collision injury mechanism. This is explained by the normalized commuters tendency of struggling and scrambling to secure a bus seat especially in rush hours [9]. In addition, this study further presents some statistical evidence for the association between the non-collision injuries and the injured body part specifically lower and upper limbs. The present findings seem to be consistent with other researches which found a statistically significant association between non-collision injuries and lower and upper limbs [6, 17]. On other hand, the current results suggest a statistical significant association between commuters in standing and walking positions when they experience non-collision injuries compared to seated commuters. This is similar to other studies which established that the standings passengers have a significantly higher likelihood to experience non-collision injuries with low severity levels compared to seated passengers [18]. In addition, other studies conclude that standing passengers in public buses have significantly higher chance of experiencing non-collision due to vulnerability of their body posture during sudden breaking and bus swerving [1, 6]. Conversely, with regards to walking passengers in the bus whilst on transit, our findings differ from those obtained in Karekla and Tyler [19] that, the passengers walking in the bus while on transit are less likely to sustain injuries while on transit as they can still maintain their natural balance.

Some interesting results show that commuters travelling on weekdays had a higher chance of experiencing non-collision injuries. In the same token, our findings accord with observations made by Strathman and colleagues that both collision and non-collision injuries among travellers tends to decrease on weekends due to less traffic volumes compared to the trend of injuries which occurs on weekdays [20].

Lastly, the study established a statistically significant association between prevalence of non-collision injuries and commuters who board the bus when it is already full. These results support Halpern et al. study [4] who observed that, buses used in public transport allow for more standing space which forces passengers to board even when all seats are full [21].

### Strengths and limitation of the study

To a best of our knowledge, this is the first study to explicitly report about the non-collision injury in Tanzania for commuter using public transport. In addition, the strength relies on choice of methods to interview commuters who actively use public transport and therefore reported injuries based on their own experience. Previous studies have focused on commuters who were involved in public transport in collision injuries with non-collision injuries given a very little attention [22]. It is worth noting that, this study aligns with goal 11 of Sustainable Development Goals (SDGs) which emphasise the need to make cities and human settlements inclusive, safe, resilient and sustainable. This study was limited to interviewing the available commuters currently using the public transport. These were selected based on their availability for commuting at the time of sampling. The injuries recorded were self-reported and might be subjected to recall bias and reporting bias. The study was limited to commuters travelling with *“daladala”* only, and therefore did not include commuters travelling with Bus Rapid Transit (BRT). Noises in buses and road bumps made the interview process a challenge to capture the interviewees’ attention and standpoint. Further, due to time and financial constraints, the study could not explore the magnitude and contribution of social factors such as income and social class of commuters to non-collision injuries. As such future studies may be carried out to address these factors. Furthermore, comparing our findings with the studies conducted in the developed countries posed a challenge because of the marked differences in the public transportation systems between the two settings.

## Conclusion

This study has demonstrated a higher prevalence of non-collision injuries among commuters using public transport in Dar es Salaam. Majority of non-collision injuries occurred between 05:00 pm to 10:00 pm on weekdays, particularly when commuters scramble to board and disembark the bus. Boarding with a full or overcrowded bus was significantly associated with higher odds of sustaining non-collision injuries. Upper and lower limbs were the most affected body parts during non-collision injuries among commuters. This paper recommends for authorities to introduce a policy that would restrict commuters or passengers to the available seats and improve the infrastructure for the bus stops and ensure that passengers only board a bus in a queue. Besides, responsible authorities should arrange with stakeholders to increasing the number of buses operating to and from the city centre. Future studies should focus on larger societal level factors such as investment in public transport, policies related to public transport and urban planning. Further studies may also be carried out to ascertain income and economic factors as individual factors that may also influence choice mode of transportation.

## Data Availability

The datasets used and/or analyzed during the current study available from the corresponding author on reasonable request.

## Abbreviations

BRT: Bus Rapid Transit
CI: Confidence Interval
LATRA: Land Transport Regulatory Authority
OR: Odds Ratio
SDG’s: Sustainable Development Goals
SUMATRA: Surface and Marine Transport Regulatory Authority
TANROADS: Tanzania Road Agency
